# The Roderick Memorial at Birmingham

**Published:** 1914-12-05

**Authors:** 


### The Roderick Memorial at Birmingham.

HOW THE VOLUNTARY PRINCIPLE WAS SAVED.

A bronze memorial tablet to the late Mr. John Roderick was unveiled at the Birmingham General Hospital on Thursday last week by Mrs. J. R. H. Smyth, wife of the chairman of the board of management. The tablet, placed in the entrance corridor of the institution, is the work of Mr. Albert Toft, and contains the bust of the late Mr. Roderick in high relief, and is framed in panels containing allegorical figures symbolising charity.

It bears the following inscription: "In memory of John Roderick, for ten years a member of the Board of Management and a munificent benefactor to this hospital. During his life he and members of his family endowed three beds, and also bequeathed the sum of over £100,000 for the benefit of this institution. He died on the 24th November, 1911."

Full tribute was paid to Mr. Roderick's munificent benefactions to the hospital by the chairman of the board, Mr. J. R. H. Smyth, Mr. J. B. Clarke, Mr. A. Pointon, and others. Mr. Gilbert Barling, senior surgeon, said that if the hospital had not received this large sum of money they were within measurable distance of no longer being a voluntary institution, and would have had to appeal to the municipality or to the Board of Guardians for assistance, because those most interested in the institution felt that they had better be rate-supported and do their work efficiently than exist under a voluntary system which, by starving them, did not allow them to do it as it ought to be done. The benefaction, however, had enabled them to make a number of necessary alterations in the arrangement of the wards, the formation of balconies where patients could have the fresh-air treatment, the re-arrangement of the dining rooms for the nursing staff and the servants, and, more important still, the provision of what they regarded as absolutely essential—a department of clinical pathology. This department would cost £3,000 or £4,000 to build, and would add an annual expenditure of £700 or £800, and when this was established they were confident that the hospital would be second to none in the kingdom. It is hoped that the department will be in full working within three months.

